# Evaluation of a Newly Developed Smartphone App for Risk Factor Management in Young Patients With Ischemic Stroke: A Pilot Study

**DOI:** 10.3389/fneur.2021.791545

**Published:** 2022-01-05

**Authors:** Viktoria Fruhwirth, Lisa Berger, Thomas Gattringer, Simon Fandler-Höfler, Markus Kneihsl, Andreas Schwerdtfeger, Elisabeth Margarete Weiss, Christian Enzinger, Daniela Pinter

**Affiliations:** ^1^Department of Neurology, Medical University of Graz, Graz, Austria; ^2^Research Unit for Neuronal Plasticity and Repair, Department of Neurology, Medical University of Graz, Graz, Austria; ^3^Department of Neuropsychology - Neuroimaging, Institute of Psychology, University of Graz, Graz, Austria; ^4^Division of Neuroradiology, Vascular and Interventional Radiology, Department of Radiology, Medical University of Graz, Graz, Austria; ^5^Department of Health Psychology, Institute of Psychology, University of Graz, Graz, Austria; ^6^Department of Clinical Psychology, Institute of Psychology, University of Innsbruck, Innsbruck, Austria

**Keywords:** stroke, secondary prevention, smartphone, app, risk factor management

## Abstract

**Background:** Efficient treatment of modifiable vascular risk factors decreases reoccurrence of ischemic stroke, which is of uttermost importance in younger patients. In this longitudinal pilot study, we thus assessed the effect of a newly developed smartphone app for risk factor management in such a cohort.

**Methods:** The app conveys key facts about stroke, provides motivational support for a healthy lifestyle, and a reminder function for medication intake and blood pressure measurement. Between January 2019 and February 2020, we consecutively invited patients with ischemic stroke aged between 18 and 55 years to participate. Patients in the intervention group used the app between hospital discharge and 3-month follow-up. The control group received standard clinical care. Modifiable risk factors (physical activity, nutrition, alcohol consumption, smoking behavior, obesity, and hypertension) were assessed during the initial hospital stay and at a dedicated stroke outpatient department three months post-stroke.

**Results:** The study cohort comprised 21 patients in the app intervention group (62% male; age = 41 ± 11 years; education = 12 ± 3 years) and 21 sex-, age- and education-matched control patients with a comparable stroke risk factor profile. Baseline stroke severity was comparable between groups (intervention: *median* NIHSS = 3; control: *median* NIHSS = 4; *p* = 0.604). Three months post-stroke, patients in the intervention group reported to be physically almost twice as active (13 ± 9 h/week) compared to controls (7 ± 5 h/week; *p* = 0.022). More intense app usage was strongly associated with higher physical activity (*r* = 0.60, *p* = 0.005) and lower consumption of unhealthy food (*r* = −0.51, *p* = 0.023). Smoking behavior (*p* = 0.001) and hypertension (*p* = 0.003) improved in all patients. Patients in the intervention group described better self-reported health-related quality of life three months post-stroke (*p* = 0.003).

**Conclusions:** Specifically designed app interventions can be an easily to implement and cost-efficient approach to promote a healthier lifestyle in younger patients with a stroke.

## Introduction

Stroke is the leading cause of long-term disability in adults (1), affecting multiple domains such as cognition, motor function, and speech (2, 3). About 10–15% of patients with stroke are so-called young patients with stroke, i.e., aged between 18 and 50 or 55 years, and the frequency of stroke within this age range is rising (4, 5).

Most young patients with stroke have a remaining life expectancy of decades and an increased risk for recurrent stroke (6). Therefore, secondary stroke prevention (e.g., medication adherence and management of modifiable risk factors) is particularly crucial within this specific stroke population (7–9).

A recent observational study in 1,730 representative patients with stroke reported an alarmingly high proportion of patients with at least one inadequately treated risk condition (up to 95% when considering vascular and lifestyle risk factors) (10). Analysis showed that adequate control of the five most relevant risk factors (hypertension, hypercholesterolemia, atrial fibrillation, smoking, and overweight) would have averted approximately half recurrent stroke events (10).

An easily to implement and cost-efficient approach to support risk factor management, especially in younger patients with stroke might be mobile health (mHealth) (11–13). The mHealth is defined as the use of mobile and wireless technologies, such as smartphones and tablets, to support the achievement of health objectives. In Western societies, more than 90% of adults aged between 18 and 54 years use smartphones (14). Due to this wide distribution among young adults and constant availability in daily routine, mHealth seems promising to support young patients with stroke in their personal risk factor self-management.

Although there is a huge amount of apps available supporting healthy living (e.g., fitness apps, nutrition apps, and smoking cessation apps) (15), surprisingly little is known regarding the benefits of such apps in stroke patient care (16). The few, yet promising, studies evaluating interventions by smartphone or tablet devices in patients with stroke showed improvements in vascular risk factors [arterial hypertension (17, 18), hypercholesterolemia (19), diabetes mellitus (19, 20)] and in lifestyle factors [physical activity (21) and obesity (20)]. In addition, these studies demonstrated feasibility and showed high patient satisfaction (22–25). However, most interventions targeted primarily either medication adherence (17–20, 26) or motivational support for a healthy lifestyle (21) or stroke education (27), rather than providing a holistic approach. Furthermore, none of these studies investigated the benefit of nutrition apps in patients with stroke, even though unhealthy nutrition was found to be a major lifestyle risk factor for stroke (7).

For this pilot study, we developed a smartphone app for secondary stroke prevention combining motivational support for a healthy lifestyle, medication adherence, and stroke education. The primary goal of this study was to investigate the effectiveness of 3 months of app usage on risk factor management in acute young patients with ischemic stroke. As secondary outcomes, we investigated clinical, cognitive, and patient-reported stroke outcomes 3 months post-stroke and patient satisfaction with the app.

## Methods

### Study Design

We conducted a pilot prospective study with a two group (intervention vs. control) pre-post intervention design to examine the effectiveness of a newly developed smartphone app for risk factor management in young patients with ischemic stroke. All patients received a detailed neurological examination and an extensive neuropsychological assessment during the initial stay at our department due to the acute event and at a pre-specified 3-month follow-up. Patients in the intervention group used a newly developed smartphone app for risk factor management between hospital discharge and the 3-month follow-up. Patients in the control group received no particular intervention between hospital discharge and the 3-month follow-up. Primary outcome measures were modifiable stroke risk factors (physical activity, nutrition, alcohol consumption, smoking behavior, obesity, and hypertension). Secondary outcome measures were clinical (stroke severity), cognitive, and patient-reported stroke outcomes 3 months post-stroke and patient satisfaction with the app. We conducted group comparisons between intervention and control group and evaluated the influence of the intensity of the app usage on the primary outcomes. This prospective study was approved by the ethics committee of the Medical University of Graz (permit number 29–494 ex 16/17). All participants gave written informed consent.

### Patients

**Figure 1:**
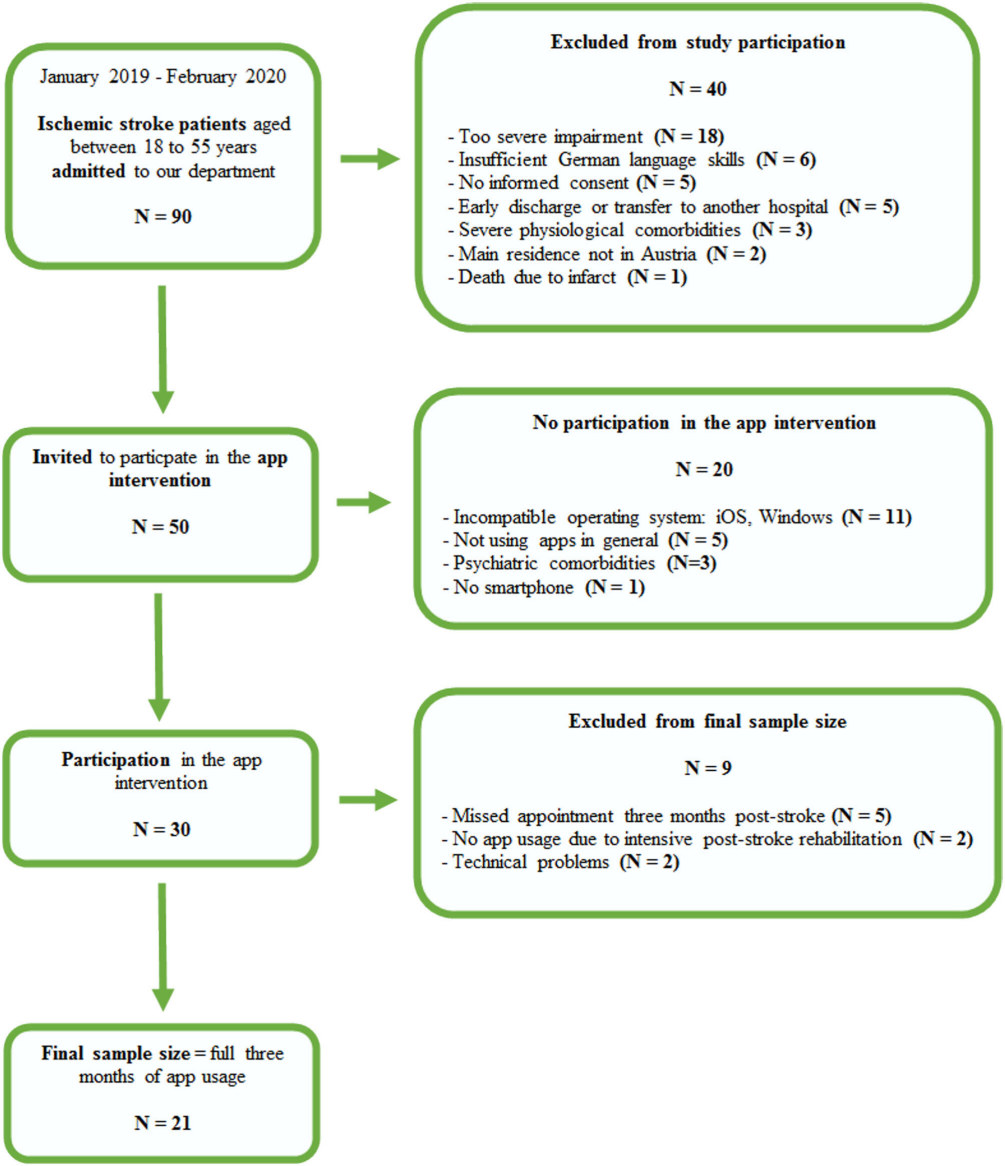
Recruitment process of the app intervention group between January 2019 and February 2020.

Consecutive patients aged between 18 and 55 years admitted to the Department of Neurology at the University Hospital Graz between January 2019 and February 2020 with an acute imaging-proven ischemic stroke were invited to participate in this prospective study. Exclusion criteria were severe pre-existing cognitive impairment or higher-order brain dysfunction precluding full engagement with the study protocol, severe dysarthria, apraxia, neglect or aphasia, insufficient German language skills, and severe impairment in fine motor skills. Patients who owned a smartphone with a compatible operating system for our app (Android) and were familiar with usage of apps were assigned to the app intervention group (Figure 1). Patients who owned a smartphone with an incompatible operating system (IOS, Windows), did not own a smartphone, did not know how to use apps, or were recruited after February 2020 (temporary app availability until February 2020) were considered as potential control patients receiving standard clinical and neuropsychological care. From all potential control patients, we created a control group that matched the intervention group regarding sex, age, education, baseline stroke severity, and stroke risk factor profile.

### Clinical and Neuropsychological Assessment

All patients underwent routine neurological examination [including assessment of stroke severity according to the National Institutes of Health Stroke Scale (NIHSS) score (range 0–42 with higher values indicating greater stroke severity), and modified Rankin Scale (mRS) score (range 0–6 with higher values indicating greater impairment)] had a vascular and lifestyle risk factor assessment and an extensive neuropsychological examination at baseline and 3 months post-stroke.

Experienced stroke physicians (TG, SF-H, and MK) assessed vascular risk factors according to medical history and clinical findings, including hypertension (blood pressure ≥ 140/90), diabetes mellitus (HbA1c ≥ 6.5%), dyslipidemia (low-density lipoprotein ≥ 100 mg/dl), atrial fibrillation, and coronary heart disease. We measured blood pressure with a standardized blood pressure monitor (MEDISANA BU 510, Neuss, Germany).

We assessed lifestyle risk factors during the neuropsychological examination (VF and LB) *via* standardized semi-structured interviews [alcohol consumption (5-point Likert scale with higher values indicating more alcohol consumption), smoking behavior (cigarettes per day), and obesity (body mass index (BMI) > 30)] and standardized questionnaires. Physical activity (average number of h/week) was assessed with the German questionnaire *Freiburger Fragebogen zur körperlichen Aktivität* (FFKA) (28). In the *Nutrition questionnaire*, patients were asked to rate on a 5-point Likert scale how often they usually consume servings of the following food groups: fruits, vegetables, legumes, whole meal foods, refined grain foods, meat, fish, dessert/sweet snacks, sugar sweetened drinks, deep fried food, dairy food, eggs, tofu/soybean curd, and alcohol. The five possible responses were: 1 = “never or rarely (<1×/week),” 2 = “about 1 serving each week,” 3 = “several servings each week,” 4 = “1–2 servings each day,” and 5 = “3 or more servings each day” (29). For the interpretation of nutrition behavior, we summarized fruits, vegetables, and legumes as “healthy nutrition” and dessert/sweets, sugar-sweetened drinks, and deep-fried food as “unhealthy nutrition.” Higher scores in the category “healthy nutrition” and lower scores in the category “unhealthy nutrition” indicate a healthier eating behavior.

Detailed information on the cognitive test battery can be found in a previous publication (3). We assessed quality of life using the EuroQol Five Dimensions questionnaire (EQ-5D; ranging from 0 to 100 with higher values indicating a better self-reported health-related quality of life) (30). Anxiety and depression were assessed with the hospital anxiety and depression scale (HADS-D; ranging from 0 to 21, values ≤ 7 are considered as clinically normal) (31). Fine motor skills were assessed for both the dominant and non-dominant hand with the Nine-Hole Peg Test (NHPT), with longer duration times indicating worse performance (32).

In addition to the standard clinical and neuropsychological assessment, patients with stroke in the app intervention group filled out a specifically created questionnaire regarding their expectations about the helpfulness of the app in terms of life style changes at baseline and an *evaluation questionnaire* (e.g., amount of app usage, content-related satisfaction, comprehensibility, and navigation) 3 months post-stroke. The baseline questionnaire consisted of 12 and the evaluation questionnaire of 24 standardized questions that patients rated on a 5-point Likert scale, ranging from “1—do not agree at all” to “5—strongly agree.” In the evaluation questionnaire, patients were also asked how many minutes per day they used the app on average to assess the self-reported intensity of app usage.

### PRESTRO: Prevent Stroke App Intervention

**Figure 2:**
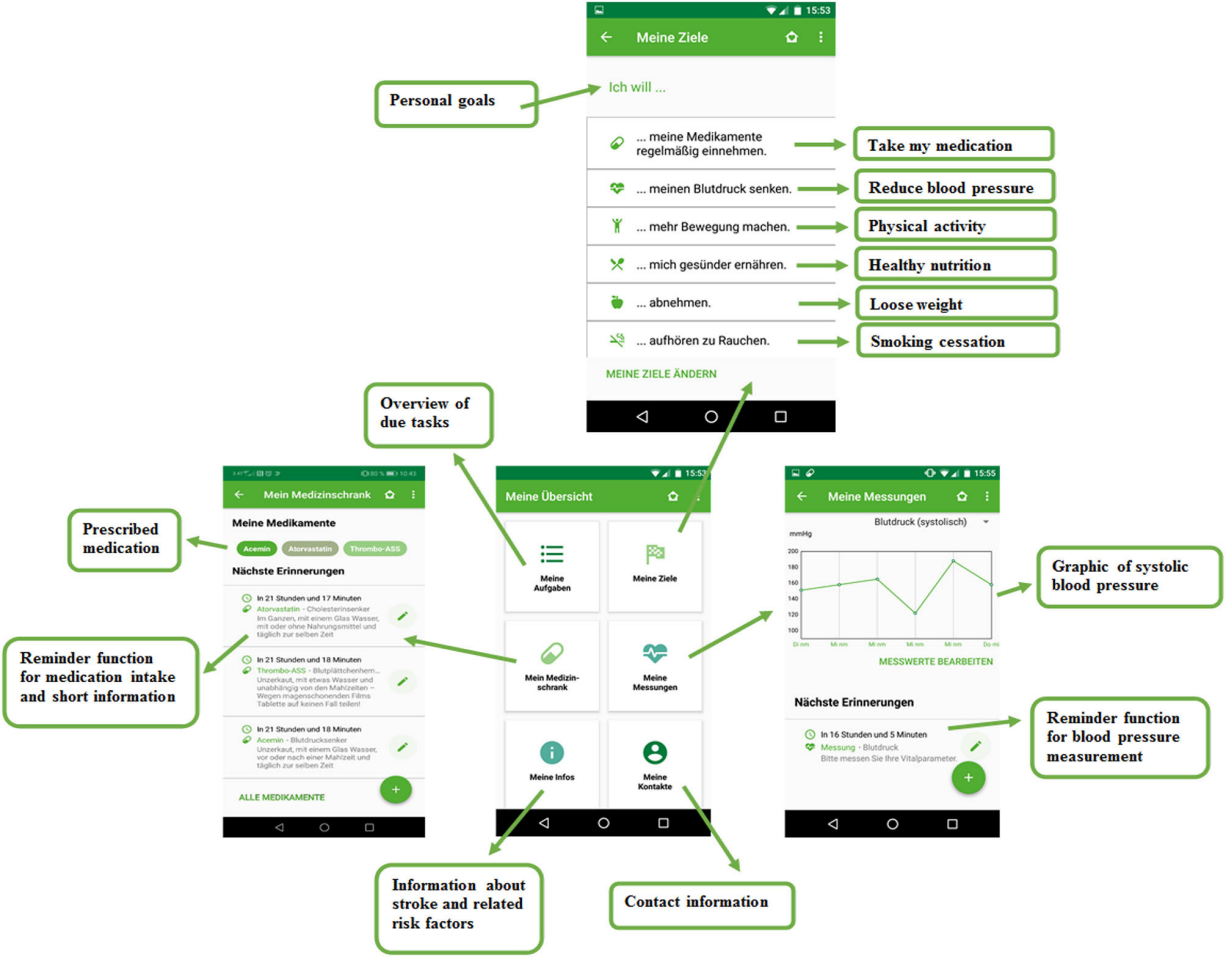
Example screenshots from our newly developed smartphone app for secondary stroke prevention.

PRESTRO—Prevent stroke is a scientifically guided smartphone app for secondary prevention after stroke, developed by a multidisciplinary team of neuropsychologists and neurologists from the Medical University Graz in cooperation with the Department of Health Psychology of Graz University and the software development company Evolaris, Graz, Austria. It was available in the *Google Play Store* between January 2019 and February 2020. The PRESTRO app combines motivational support for a healthy lifestyle (physical activity, healthy nutrition, and smoking cessation), a reminder function for medication intake and blood pressure measurement and stroke education in a comprehensible format (Figure 2).

Based on psychological theories, such as self-efficacy expectation (33), action planning (34), reinforcement learning (35), building of habits (36), motivation (37), and SMART goals (38), we created 42 tips for each lifestyle factor: physical activity, healthy nutrition, and smoking cessation. By selecting the corresponding risk factors, patients get three tips for increasing physical activity and three tips for healthy nutrition per week *via* push notifications. A push notification is a message directly sent from the app that appears on the display, independent of current app or smartphone usage. This way, the app functions as a “personal trainer,” providing daily reminding, motivation, and concrete ideas how to implement healthy lifestyle in one's personal routine. To actively involve patients, they were asked to define personal goals regarding physical activity and healthy nutrition once a week. At the end of each week, patients were asked to evaluate the degree of goal achievement and were praised for successful completion and further encouraged when not completing their goals.

Furthermore, the PRESTRO app provides a virtual medicine cabinet that includes commonly prescribed antiplatelet drugs, anticoagulants, antihypertensives, cholesterol-lowering agents, and antidiabetic drugs. Patients with stroke can select their prescribed medication and get information on intake recommendation, effect of the medication, and an explanation on how the medication can help to prevent a further stroke. At individually chosen time points, patients get a reminder from the app to take their medication, which has shown to substantially improve medication adherence in patients with stroke (17, 26). After medication intake, patients can tick off the due medication task in the app. Similarly, the app reminds patients to measure and document their systolic and diastolic blood pressure, pulse, and weight. The app displays the measured values graphically in a comprehensible format (Figure 2).

The educational part of the app provides key facts about the origin and development of an ischemic stroke and its associated risk factors. Previous studies have shown that psychoeducation promotes active coping with the disease (39) and enhances knowledge (40) and self-efficacy in patients with stroke (41, 42).

Patients who fulfilled the app-intervention inclusion criteria received the login details and a detailed booklet containing information on the provided app features and navigation during their initial hospital stay. Two days after installing the app, the implementing psychologists (VF and LB) revisited the patient and made sure that the app was working correctly, and there were no further questions. Patients were instructed to use the app for 3 months.

### Statistical Analyses

Demographics, clinical, and neuropsychological scores were analyzed with IBM SPSS Statistics 26 (IBM Corp., Armonk, NY, USA). The level of significance was set at 5%. Normal distribution was assessed with the Kolmogorov–Smirnov test and *via* skewness and kurtosis. Outliers were assessed *via* boxplots.

Comparisons between the app intervention and control group were conducted with unpaired *t*-tests (for normally distributed continuous variables) and Mann–Whitney *U*-tests (for non-normally distributed variables). Longitudinal within- and between-group comparisons were done with ANOVA. Correlation analysis was performed with Spearman (ordinal data) and Pearson (metric data) correlations.

## Results

### Patient Selection and Characteristics

The recruitment process for the app intervention group is shown in Figure 1. From the initially invited 50 young patients with ischemic stroke, 60% were willing to participate in the app intervention. The main reason for not participating in the app intervention was incompatible operating systems (*N* = 11). Only one potential participant did not own a smartphone. The control group consisted of sex-, age- and education-matched patients with acute ischemic stroke with a comparable stroke risk factor profile who owned a smartphone with an incompatible operating system (IOS, Windows) or had technical problems (*N* = 9), did not own a smartphone (*N* = 1), did not use apps in general (*N* = 5), or were recruited after February 2020 (*N* = 6).

The final sample comprised 21 young patients with ischemic stroke in the app intervention group and 21 patients in the control group. At baseline, the app intervention and control group did not significantly differ regarding age, sex, education, stroke severity, post-stroke rehabilitation therapy, stroke risk factors, and fine motor skills (Table 1). The most prevalent stroke risk factors were hypertension, hyperlipidemia, and smoking. The mRS at discharge was slightly higher in the control group compared to the intervention group. In addition, patients in the control group had higher anxiety scores and performed worse in the cognitive screening. However, there were no significant group differences in all other cognitive tasks. Regarding stroke severity, cognition, and fine motor skills, successful smartphone usage would have been possible in all included patients.

**Table 1:** Demographics, clinical, and neuropsychological characteristics of young ischemic stroke patients in the app intervention group and control group at stroke onset.

	**App intervention group *N* = 21**	**Control group *N* = 21**	***P*-value**
**Demographics**			
Age in years, *mean* (*SD*); *median* (*IQR*); *min*–*max*	41 (11); 45 (30–50); 20–55	47 (8); 50 (44–53); 25–54	0.056
Sex, female, *N* (%)	8 (38.1)	9 (42.9)	0.760
Education in years, *mean* (*SD*); *median* (*IQR*); *min*–*max*	12 (3); 11 (10–14); 9–19	11 (3); 10 (10–13); 8–18	0.341
**Clinical characteristics**			
NIHSS, *median* (*IQR*)	3 (1–6)	4 (1–7)	0.604
mRS at discharge, *median* (*IQR*)	1 (0–2)	2 (1–2)	**0.008**
Rehabilitation therapy after discharge, *N* (%)	10 (47.6)	13 (61.9)	0.365
**Vascular risk factors**			
Hypertension, *N* (%)	8 (38.1)	8 (38.1)	0.999
Systolic blood pressure, *mean* (*SD*); *median* (*IQR*); *min*–max^*^	139 (19); 133 (123–156); 115–190	140 (22); 141 (128–156); 100–190	0.855
Diastolic blood pressure, *mean* (*SD*); *median* (*IQR*); *min*–max^*^	87 (11); 88 (79–96); 70–108	83 (13); 83 (73–93); 58–107	0.236
Diabetes mellitus, *N* (%)	0 (0)	2 (9.5)	0.162
Hyperlipidemia, *N* (%)	9 (42.9)	5 (23.8)	0.200
Atrial fibrillation, *N* (%)	1 (4.8)	0 (0)	0.329
Coronary heart disease, *N* (%)	0 (0)	1 (4.8)	0.329
**Lifestyle and psychological risk factors**			
Active smoking, *N* (%)	6 (28.6)	8 (38.1)	0.524
Clinically diagnosed alcohol abuse (ICD-10 criterion F10.2), *N* (%)	3 (14.3)	1 (4.8)	0.306
Self-reported alcohol consumption, *mean* (SD); *median* (*IQR*); *min*–max^**^	1.9 (1.0); 2.0 (1.0–2.0); 1–4	1.5 (0.8); 1.0 (1.0–2.0); 1–3	0.160
Weight in kg, *mean* (*SD*); *median* (*IQR*); *min*–*max*	84 (16); 84 (70–100); 55–110	80 (14); 82 (69–90); 56–105	0.497
BMI, *mean* (*SD*); *median* (*IQR*); *min*–*max*	28 (5); 27 (24–32); 19–34	27 (3); 27 (24–29); 23–32	0.499
Physical activity in h/week, *mean* (SD); *median* (*IQR*); *min*–*max*	7 (6); 6 (2–11); 0–24	5 (4); 4 (2–7); 0–17	0.272
Self-reported healthy nutrition, *mean* (*SD*); *median* (*IQR*); *min*–max^***^	2.7 (0.7); 2.7 (2.2–3.2); 1–4	2.8 (0.7); 3.0 (2.5–3.1); 1–4	0.526
Self-reported unhealthy nutrition, *mean* (*SD*); *median* (*IQR*); *min*–max^***^	2.5 (0.7); 2.7 (2.0–3.0); 1–4	2.5 (0.5); 2.7 (2.2–2.8); 1–3	0.865
HADS-D Depression, *mean* (*SD*); *median* (*IQR*); *min*–max^*^	3 (4); 2 (1–5); 0–12	5 (3); 5 (2–8); 0–12	0.176
HADS-D Anxiety, *mean* (*SD*); *median* (*IQR*); *min*–max^*^	5 (3); 4 (3–7); 1–13	8 (3); 8 (5–10); 1–15	**0.014**
**Medication**, ***N*** **(%)**			
Antiplatelets	18 (85.7)	17 (81.0)	0.688
Anticoagulants	2 (9.5)	4 (19.0)	0.390
Antihypertensives	8 (38.1)	8 (38.1)	0.999
Cholesterol-lowering	11 (52.4)	10 (47.6)	0.765
Antidiabetics	0 (0.0)	1 (4.8)	0.329
Antidepressants	3 (14.3)	7 (33.3)	0.155
**Neuropsychological assessment**, ***mean*** **(*****SD*****);** ***median*** **(*****IQR*****);** ***min***–***max***			
EQ-5D self-reported quality of life	70 (18); 70 (53–88); 33–99	59 (25); 50 (40–83); 0–100	0.097
MoCA (raw score)	28 (2); 29 (28–30); 25–30	25 (5); 27 (21–28); 13–30	**0.003**
SDMT (z-norm)	−1.0 (1.2); −1.0 (−1.8–(−0.5)); −3.0–1.5	−1.5 (1.4); −1.5 (−3.0–0.0); −3.0–1.5	0.233
CTMT-2 (t-norm)^*^	44 (10); 43 (36–54); 26–61	40 (14); 39 (31–50); 18–72	0.334
CTMT-5 (t-norm)^†^	39 (10); 39 (30–46); 22–61	37 (16); 37 (25–48); 18–68	0.744
NHPT in seconds (dominant hand^‡^	25 (8); 22 (20–27); 19–42	25 (7); 23 (20–28); 15–45	0.832

*N, sample size; IQR, interquartile range; NIHSS, National Institutes of Health Stroke Scale; mRS, modified Rankin Scale; EQ-5D, EuroQol Five Dimensions (health-related quality of life); HADS-D, hospital anxiety and depression scale; MoCA, Montreal Cognitive Assessment (cognitive screening); SDMT, Symbol Digital Modalities Test (processing speed); CTMT, Comprehensive Trail Making Test (subtest 2: attention, subtest 5: cognitive flexibility); NHPT, Nine-Hole Peg Test (fine motor skills);*

* *missing in one patient;*

** *Self-rating on a 5-point Likert scale (1–5) where higher values indicate more alcohol consumption;*

*** *higher scores in “healthy nutrition” and lower scores in “unhealthy nutrition” indicate a healthier eating behavior;*

† *missing in two patients;*

‡ *missing in three patients. Bold values indicate significant p-values*.

### Primary Outcome: Beneficial Effect of the PRESTO App on Stroke Risk Factor Management

The primary outcome measures (physical activity, nutrition, alcohol consumption, smoking behavior, obesity, and hypertension) are presented for the intervention and control group 3 months post-stroke in Table 2.

**Table 2:** Primary outcome: Modifiable stroke risk factors for the intervention and control group 3 months post-stroke.

	**App intervention group *N* = 21**	**Control group *N* = 21**	***P*-value**	***Cohen's d***
Physical activity in h/week, *mean* (SD); *median* (*IQR*); *min*–max^*^	13 (9); 11 (8–17); 0–41	7 (5); 8 (4−10); 0–16	**0.022**	**0.7**
Self-reported healthy nutrition, *mean* (*SD*); *median* (*IQR*); *min*–*max*^†^^***^	3.2 (0.6); 3.3 (3.0–3.5); 1–4	3.2 (0.5); 3.0 (3.0–3.7); 2–4	0.996	0.0
Self-reported unhealthy nutrition, *mean* (*SD*); *median* (*IQR*); *min*–max^*^^***^	2.0 (0.7); 2.0 (1.5–2.3); 1–4	2.0 (0.4); 2.0 (1.8–2.3); 1–3	0.855	0.1
Self-reported alcohol consumption, *mean* (SD); *median* (*IQR*); *min*–max^***^	1.6 (0.7); 1.0 (1.0–2.0); 1–3	1.2 (0.4); 1.0 (1.0–1.0); 1–2	**0.015**	**0.8**
Active smoking, *N* (%)	1 (4.8)	2 (9.5)	0.560	0.2
Systolic blood pressure, *mean* (*SD*); *median* (*IQR*); *min*–*max*^†^	128 (11); 130 (120–137); 101–148	128 (16); 129 (115–140); 99–159	0.875	0.1
Diastolic blood pressure, *mean* (*SD*); *median* (*IQR*); *min*–*max*^†^	85 (14); 83 (78–93); 56–113	82 (9); 82 (72–88); 69–104	0.481	0.2
Weight in kg, *mean* (*SD*); *median* (*IQR*); *min*–*max*^†^	81 (16); 81 (68–93); 55–111	80 (13); 81 (69–92); 59–105	0.832	0.1
BMI, *mean* (*SD*); *median* (*IQR*); *min*–*max*^†^	27 (5); 27 (23–32); 19–35	27 (3); 26 (25–30); 22–32	0.893	0.0

*N, sample size; IQR, interquartile range; BMI, body mass index*.

* *missing in one patient;*

** Self-rating on a 5-point Likert scale (1–5) where higher values indicate more alcohol consumption;

*** *higher scores in “healthy nutrition” and lower scores in “unhealthy nutrition” indicate a healthier eating behavior;*

† *missing in two patients. Bold values indicate significant p-values*.

### Physical Activity

Baseline self-reported physical activity was comparable between the app intervention (7 ± 6 h/week) and the control group (5 ± 4 h/week; *p* = 0.272). However, 3 months post-stroke, app users were physically almost twice as active (13 ± 9 h/week) compared to controls (7 ± 5 h/week; *p* = 0.022). Within the app intervention group, a more intense app usage was strongly associated with higher physical activity (*r* = 0.60, *p* = 0.005). Physical activity 3 months post-stroke was neither associated with mRS at discharge (*p* = 0.311) nor with mRS 3 months post-stroke (*p* = 0.109). In one patient with mRS = 4 (control group), physical activity was not assessed at follow-up and therefore not included in the analyses.

### Nutrition

All young patients with ischemic stroke improved their nutrition 3 months post-stroke independent of app-usage. ANOVAs revealed an increase in self-reported healthy nutrition (*p* < 0.001) and a decrease in self-reported unhealthy nutrition (*p* < 0.001). However, considering the intensity of the app usage in the app intervention group, we found a strong association between more intense app usage and lower consumption of unhealthy food 3 months post-stroke (*r* = −0.51, *p* = 0.023).

### Alcohol Consumption

Self-reported alcohol consumption decreased in all young patients with ischemic stroke 3 months post-stroke (*p* = 0.041). However, patients in the app intervention group reported to consume more alcohol than patients in the control group 3 months post-stroke (*p* = 0.015).

### Smoking Behavior

Smoking behavior also improved in all young patients with ischemic stroke 3 months post-stroke (*p* = 0.001). At baseline, six young patients with ischemic stroke in the app intervention group and eight patients in the control group smoked. Three-months post-stroke, only one patient in the app intervention group and two patients in the control group did not quit smoking.

### Obesity and Hypertension

Mean systolic blood pressure (intervention: *p* = 0.003; control: *p* = 0.033) improved in all young patients with ischemic stroke independent of app usage 3 months post-stroke. Mean diastolic blood pressure (intervention: *p* = 0.396; control: *p* = 0.692), weight (intervention: *p* = 0.131; control: *p* = 0.983), and BMI (intervention: *p* = 0.127; control: *p* = 0.833) did not change within 3 months post-stroke.

When correcting for baseline group differences in the Montreal Cognitive Assessment (MoCA), anxiety, and mRS at discharge, the group difference in the self-reported alcohol consumption 3 months post-stroke is no longer significant (*p* = 0.222). All other primary results remain stable.

### Secondary Outcome 1: Clinical, Cognitive, and Patient-Reported Stroke Outcome

Clinical and neuropsychological characteristics 3 months post-stroke are presented in Table 3 (baseline characteristics in Table 1). Baseline stroke severity (NIHSS) was comparable between young patients with ischemic stroke in the app intervention group and control patients. However, 3 months post-stroke, the degree of disability or dependence in daily activities (mRS) in the app intervention group was significantly lower than in the control group. Similar, self-reported quality of life was higher in the app intervention group than in the control group 3 months post-stroke, despite comparable baseline values. Interestingly, participating in the app intervention group was associated with better self-reported quality of life 3 months post-stroke independent of baseline stroke severity (*p* = 0.003). Cognitive flexibility was higher in the app intervention group 3 months post-stroke than in the control group, despite comparable baseline performance (*p* = 0.039).

**Table 3:** Secondary outcome: Clinical and neuropsychological characteristics of young patients with ischemic stroke in the app intervention group and control group 3 months post-stroke.

	**App intervention group *N* = 21**	**Control group *N* = 21**	***P*-value**	***Cohen's d***
**Clinical characteristics**, ***median*** **(*****IQR*****)**				
NIHSS	0 (0–0)	1 (0–2)	**0.005**	**0.9**
mRS	0 (0–1)	2 (1-2)	**0.008**	**0.9**
**Neuropsychological assessment**, ***mean*** **(*****SD*****);** ***median*** **(*****IQR*****);** ***min***–***max***				
EQ-5D self-reported quality of life^*^	84 (15); 90 (73–95); 50-100	72 (17); 77 (60-80); 30-100	**0.030**	**0.7**
HADS-D Depression^*^	2 (2); 1 (0–2); 0–7	3 (4); 2 (1–6); 0-13	0.066	0.6
HADS-D Anxiety^*^	3 (3); 3 (1–5); 0–12	5 (3); 6 (3–8); 0–12	**0.041**	**0.7**
MoCA (raw score)	29 (2); 29 (28–30); 24–30	27 (3); 29 (26–29); 20–30	0.122	0.5
SDMT (z-norm)	−0.1 (1.0); −0.3 (−0.5–0.5); −2.0–2.0	−0.7 (1.2); −0.5 (−1.7–0.5); −2.8–1.5	0.075	0.6
CTMT-2 (t-norm)	52 (12); 54 (42–60); 36–76	44 (17); 41 (36–48); 18–85	0.088	0.5
CTMT-5 (t-norm)	49 (11); 46 (43–56); 36–83	40 (15); 41 (28–49); 18–71	**0.039**	**0.7**

*N, sample size; IQR, interquartile range; NIHSS, National Institutes of Health Stroke Scale; mRS, modified Rankin Scale; SD, Standard deviation; EQ-5D, EuroQol Five Dimensions (health-related quality of life); HADS-D, Hospital anxiety and depression scale; MoCA, Montreal Cognitive Assessment (cognitive screening); SDMT, Symbol Digital Modalities Test (processing speed); CTMT, Comprehensive Trail Making Test (subtest 2: attention, subtest 5: cognitive flexibility)*.

* *missing in one patient. Bold values indicate significant p-values*.

When correcting for baseline group differences in the MoCA, anxiety, and mRS at discharge, the group differences in the NIHSS (*p* = 0.411), mRS (*p* = 0.942), self-reported health-related quality of life (*p* = 0.920), anxiety (*p* = 0.278), and cognitive flexibility (*p* = 0.878) 3 months post-stroke are no longer significant.

### Secondary Outcome 2: Patient Satisfaction With the PRESTRO App

Patient satisfaction with the PRESTRO app was high. About 90% of young patients with ischemic stroke in the app intervention group reported “high” or “very high” overall satisfaction regarding motivational support for healthy lifestyle, reminder function for medication intake and blood pressure measurement, and stroke education. All young patients with ischemic stroke in the intervention group rated the app as easy to operate.

## Discussion

In this pilot study, we developed and preliminary evaluated a smartphone app for secondary stroke prevention combining motivational support for a healthy lifestyle (physical activity, healthy nutrition, and smoking cessation), medication adherence, and stroke education. We evaluated the effectiveness of the app on stroke risk factor management, clinical, cognitive, and patient-reported stroke outcome 3 months post-stroke and patient satisfaction with the app in young patients with ischemic stroke. App users profited from motivational support provided by the app resulting in increased physical activity. Post-stroke disability was lower and self-reported quality of life was higher in the app intervention than in the control group 3 months after stroke. Furthermore, the majority of app users were highly satisfied with our newly developed smartphone app in terms of content and usage.

Regarding risk factor management, our smartphone app seemed to have helped young patients with ischemic stroke to promote a healthy lifestyle. Three months post-stroke, app users were physically almost twice as active compared to patients in the control group. This result is in line with a previous study (21) that demonstrated the positive effect of an app that incorporates evidence-based behavior change techniques (feedback, self-monitoring, and social support) on physical activity in patients with stroke. Furthermore, we found an association between more intense app usage and better eating habits 3 months post-stroke. Physical activity and nutrition are two crucial modifiable risk factors of stroke (7) and are strongly associated with other stroke risk factors such as obesity, diabetes mellitus, hyperlipidemia, and hypertension (43). Therefore, changing lifestyle is crucial to prevent further strokes and can be supported by an app intervention.

Despite increased physical activity and better eating habits, we found no significant improvement in weight or BMI 3 months post-stroke. A possible explanation might be the short duration of only 3 months app usage in our study. A study that conducted an app intervention in patients with stroke for 6 months found significant improvements in BMI and waist circumference (20), whereas a different study that conducted an app intervention for only 6 weeks also found no changes in BMI (21). A longer follow-up time (and a larger study sample) might be necessary to find significant changes in more resistant measures such as weight or BMI.

Studies that investigated the effect of app interventions on blood pressure measurement in patients with stroke showed inconclusive results. Some report stronger improvements in the intervention than the control group (18), whereas others found no such differences (17, 21, 26). In our study, all young patients with ischemic stroke significantly improved in systolic blood pressure independent of app usage. However, app users reported that the reminder function for blood pressure measurement was very helpful to keep track of their personal blood pressure values. Moreover, they experienced the motivational support for smoking cessation as very helpful, yet all young patients with ischemic stroke improved their smoking behavior 3 months post-stroke.

Interestingly, in addition to the beneficial effect of the app on healthy lifestyle, we found that participating in the app intervention was associated with better self-reported quality of life 3 months post-stroke independent from baseline stroke severity. The app aims to empower young patients with ischemic stroke to actively cope with the changes following stroke. By giving specific lifestyle and risk factor management advice, patients can actively prevent a further stroke. This might help patients to improve the resilience and self-efficacy of patients (44). Recent studies showed an association between higher psychological resilience and better long-term stroke outcome (45, 46). Active coping promoted by the app could improve the resilience of patients, which could lead to higher rehabilitation motivation and furthermore to better subjective health status and lower stroke severity 3 months post-stroke.

In line with a previous study demonstrating that an app intervention can improve the knowledge of patients on stroke risk factors (27), our app focuses on comprehensible stroke education. It provides key facts about the origin and development of an ischemic stroke, its associated risk factors, and ways to diminish those risk factors. Although such stroke education is part of the standard care during the initial stay, the flood of information regarding for example the disease itself, newly prescribed medication, results of additional medical examinations, and rehabilitation approaches might be quite overwhelming. This may be especially so during the acute stage, when vigilance might be reduced (47), and cognitive deficits are frequently present (3, 48). Young patients with ischemic stroke experienced the summary of the most important stroke facts provided by our app as helpful after hospital discharge.

Our study does not come without limitations. The main limitation of our study was that our patients were not randomly allocated to the intervention and control groups. Similar to a previous study (19), our app was only available for Android-operated devices, and therefore, in line with this study, we decided to use patients with different operating systems as control group. However, the vast majority of smartphone owners with a different operating system reported that they would have used the app if possible. Furthermore, it is important to notice that the lifestyle risk factors (physical activity, nutrition, and smoking behavior) were assessed *via* questionnaires and therefore might be biased due to social desirability. However, we compared an intervention and control group that were both examined with the same questionnaires, and therefore social desirability should have similar effects on response tendencies among groups. Furthermore, previous studies (49, 50) showed moderate to strong correlations between self-reported lifestyle behavior and objectively measured lifestyle behavior. In addition, despite good matching in many relevant baseline characteristics, our groups differed at baseline in the MoCA score, anxiety, and mRS at discharge. When including these variables in the analyses as covariates, the secondary outcomes are no longer significant. However, this might also be due to a statistical overfitting in a rather small sample and should be further explored in a larger sample. Furthermore, we included no intervention regarding the lifestyle risk factor alcohol, which is a known risk factor for recurrent events. We explained this risk factor in the information part of the app, highlighting that patients with an alcohol addiction need professional treatment that is difficult to provide *via* app. In addition, the sample size of this pilot study was limited, and larger (multicenter) cohorts will be necessary to further explore the beneficial effects of smartphone apps on risk factor management in patients with stroke. In further studies comprising a larger cohort, it would also be important to analyze dropout patients (i.e., intention to treat analysis) to identify baseline characteristics that possibly enhance the risk of poor compliance. Nevertheless, our study shows promising results regarding lifestyle improvement, stroke outcome, and patient satisfaction that are in line with previous studies (17, 18, 20–25).

## Conclusion

In conclusion, young patients with ischemic stroke, especially benefited from motivational support leading to increased physical activity. Furthermore, we found that participating in the app intervention was associated with better self-reported quality of life 3 months post-stroke independent of baseline stroke severity. This suggests enormous potential for such specifically designed app interventions, complementing personal clinical care.

## Data Availability Statement

Data that support the findings of this study are available from the corresponding author upon reasonable request.

## Ethics Statement

This study involved human participants and was reviewed and approved by the Ethics Committee of the Medical University of Graz (permit number 29-494 ex 16/17). The patients provided their written informed consent to participate in this study.

## Author Contributions

VF, DP, LB, TG, SF-H, and MK: data collection. VF, DP, and LB: material preparation and analysis. VF: first draft of the manuscript. All authors commented on previous versions of the manuscript, read, approved the final manuscript, design, and contributed to the study conception.

## Funding

This study was funded by the Styrian Government, Austria (Das Land Steiermark) (ABT08-119893/2017).

## Conflict of Interest

The authors declare that the research was conducted in the absence of any commercial or financial relationships that could be construed as a potential conflict of interest.

## Publisher's Note

All claims expressed in this article are solely those of the authors and do not necessarily represent those of their affiliated organizations, or those of the publisher, the editors and the reviewers. Any product that may be evaluated in this article, or claim that may be made by its manufacturer, is not guaranteed or endorsed by the publisher.

## References

[1] Grefkes C Fink GR. Recovery from stroke: current concepts and future perspectives. Neurol Res Pract. (2020) 2:17. 10.1186/s42466-020-00060-6 33324923 PMC7650109

[2] Goeggel Simonetti B Mono M-L Huynh-Do U Michel P Odier C Sztajzel R. Risk factors, aetiology and outcome of ischaemic stroke in young adults: the Swiss Young Stroke Study (SYSS). J Neurol. (2015) 262:2025–32. 10.1007/s00415-015-7805-5 26067218

[3] Pinter D Enzinger C Gattringer T Eppinger S Niederkorn K Horner S. Prevalence and short-term changes of cognitivedysfunction in young ischemic stroke patients. Eur J Neurol. (2018) 26:727–32. 10.1111/ene.13879 30489673 PMC6491967

[4] Tibæk M Dehlendorff C Jørgensen HS Forchhammer HB Johnsen SP Kammersgaard LP. Increasing incidence of hospitalization for stroke and transient ischemic attack in young adults: a registry-based study. J Am Heart Assoc. (2016) 5:1–9. 10.1161/JAHA.115.003158 27169547 PMC4889186

[5] Ekker MS Boot EM Singhal AB Tan KS Debette S Tuladhar AM. Epidemiology, aetiology, and management of ischaemic stroke in young adults. Lancet Neurol. (2018) 17:790–801. 10.1016/S1474-4422(18)30233-3 30129475

[6] Maaijwee N A. MM Rutten-Jacobs LC Schaapsmeerders P van Dijk EJ de Leeuw F-E. Ischaemic stroke in young adults: risk factors and long-term consequences. Nat Rev Neurol. (2014) 10:315–25. 10.1038/nrneurol.2014.72 24776923

[7] O'Donnell MJ Xavier D Liu L Zhang H Chin SL Rao-Melacini P. Risk factors for ischaemic and intracerebral haemorrhagic stroke in 22 countries (the INTERSTROKE study): a case-control study. Lancet. (2010) 376:112–23. 10.1016/S0140-6736(10)60834-3 20561675

[8] von Sarnowski B Putaala J Grittner U Gaertner B Schminke U Curtze S. Lifestyle risk factors for ischemic stroke and transient ischemic attack in young adults in the Stroke in Young Fabry Patients study. Stroke. (2013) 44:119–25. 10.1161/STROKEAHA.112.665190 23150649

[9] Sakakibara BM Kim AJ Eng JJ. A systematic review and meta-analysis on self-management for improving risk factor control in stroke patients. Int J Behav Med. (2017) 24. 10.1007/s12529-016-9582-7 27469998 PMC5762183

[10] Boehme C Toell T Mayer L Domig L Pechlaner R Willeit K. The dimension of preventable stroke in a large representative patient cohort. Neurology. (2019) 93:E2121–32. 10.1212/WNL.0000000000008573 31672716

[11] Grau-Pellicer M Lalanza JF Jovell-Fernández E Capdevila L. Impact of mHealth technology on adherence to healthy PA after stroke: a randomized study. Top Stroke Rehabil. (2020) 27:354–68. 10.1080/10749357.2019.1691816 31790639

[12] Burns SP Terblanche M Perea J Lillard H DeLaPena C Grinage N. mHealth intervention applications for adults living with the effects of stroke: a scoping review. Arch Rehabil Res Clin Transl. (2021) 3:100095. 10.1016/j.arrct.2020.100095 33778470 PMC7984984

[13] Krishnamurthi R Hale L Barker-Collo S Theadom A Bhattacharjee R George A. Mobile technology for primary stroke prevention. Stroke. (2019) 50:196–8. 10.1161/STROKEAHA.118.023058 30580699

[14] Berenguer A Goncalves J Hosio S Ferreira D Anagnostopoulos T Kostakos V. Are Smartphones Ubiquitous? : An in-depth survey of smartphone adoption by seniors. IEEE Consum Electron Mag. (2017) 6:104–10. 10.1109/MCE.2016.261452427295638

[15] Aitken M Clancy B Nass D. The growing value of digital health: evidence impact on human health the healthcare system. New Jersey, NJ: IQVIA Institute for Human Data Science (2017). Available online at: https://www.iqvia.com/insights/the-iqvia-institute/reports/the-growing-value-of-digital-health

[16] Fruhwirth V Enzinger C Weiss E Schwerdtfeger A Gattringer T Pinter D. Use of smartphone apps in secondary stroke prevention. Wiener Medizinische Wochenschrift. (2020) 170:41–54. 10.1007/s10354-019-00707-3 31535230

[17] Kamal AK Shaikh Q Pasha O Azam I Islam M Memon AA. A randomized controlled behavioral intervention trial to improve medication adherence in adult stroke patients with prescription tailored Short Messaging Service (SMS)-SMS4Stroke study. BMC Neurol. (2015) 15:1–11. 10.1186/s12883-015-0471-5 26486857 PMC4618367

[18] Ovbiagele B Jenkins C Patel S Brunner-Jackson B Anderson A Saulson R. Mobile health medication adherence and blood pressure control in recent stroke patients. J Neurol Sci. (2015) 358:535–7. 10.1016/j.jns.2015.10.008 26463572

[19] Requena M Montiel E Baladas M Muchada M Boned S López R. Farmalarm: application for mobile devices improves risk factor control after stroke. Stroke. (2019) 50:1819–24. 10.1161/STROKEAHA.118.024355 31167621

[20] Seo WK Kang J Jeon K Lee K Lee S Kim JH. Feasibility of using mobile applications for the monitoring and management of stroke-associated risk factors. J Clin Neurol. (2015) 11:142–8. 10.3988/jcn.2015.11.2.142 25851892 PMC4387479

[21] Paul L Wyke S Brewster S Sattar N Gill JMR Alexander G. Increasing physical activity in stroke survivors using STARFISH, an interactive mobile phone application: A pilot study. Top Stroke Rehabil. (2016) 23:170–7. 10.1080/10749357.2015.1122266 27077973

[22] Denham AMJ Halpin S Twyman L Guillaumier A Bonevski B. Prevent 2 ^nd^ Stroke: a pilot study of an online secondary prevention program for stroke survivors. Aust N Z J Public Health. (2018) 1–7. 10.1111/1753-6405.12794 29888829

[23] Kamwesiga JT Eriksson GM Tham K Fors U Ndiwalana A von Koch L. A feasibility study of a mobile phone supported family-centred ADL intervention, F@ce^TM^, after stroke in Uganda. Global Health. (2018) 14:1–13. 10.1186/s12992-018-0400-7 30111333 PMC6094578

[24] Sarfo FS Adusei N Ampofo M Kpeme FK Ovbiagele B. Pilot trial of a tele-rehab intervention to improve outcomes after stroke in Ghana: A feasibility and user satisfaction study. J Neurol Sci. (2018) 387:94–7. 10.1016/j.jns.2018.01.039 29571880 PMC5868410

[25] Sureshkumar K Murthy GVS Natarajan S Naveen C Goenka S Kuper H. Evaluation of the feasibility and acceptability of the “Care for Stroke” intervention in India, a smartphoneenabled, carer-supported, educational intervention for management of disability following stroke. BMJ Open. (2016) 6. 10.1136/bmjopen-2015-009243 26839011 PMC4746451

[26] Sarfo F Treiber F Gebregziabher M Adamu S Patel S Nichols M. PINGS (Phone-based intervention under nurse guidance after stroke) interim results of a pilot randomized controlled trial. Stroke. (2018) 49:236–9. 10.1161/STROKEAHA.117.019591 29222227 PMC5742065

[27] Kang Y-N Shen H-N Lin C-Y Elwyn G Huang S-C Wu T-F. Does a Mobile app improve patients' knowledge of stroke risk factors and health-related quality of life in patients with stroke? A randomized controlled trial. BMC Med Inform Decis Mak. (2019) 19:282. 10.1186/s12911-019-1000-z 31864348 PMC6925878

[28] Frey I Berg A Grathwohl DK Keul J. Freiburg Questionnaire of physical activity–development, evaluation and application. Soz Praventivmed. (1999) 44:55–64. 10.1007/BF01667127 10407953

[29] Stewart RAH Wallentin L Benatar J Danchin N Hagström E Held C. Dietary patterns and the risk of major adverse cardiovascular events in a global study of high-risk patients with stable coronary heart disease. Eur Heart J. (2016) 37:1993–2001. 10.1093/eurheartj/ehw125 27109584 PMC4929377

[30] Devlin NJ Brooks R. EQ-5D and the EuroQol group: past, present and future. Appl Health Econ Health Policy. (2017) 15:127. 10.1007/s40258-017-0310-5 28194657 PMC5343080

[31] Beekman E Verhagen A. Clinimetrics: Hospital anxiety and depression scale. J Physiother. (2018) 64:198. 10.1016/j.jphys.2018.04.003 29895416

[32] Grice KO Vogel KA Le V Mitchell A Muniz S Vollmer MA. Adult norms for a commercially available nine hole peg test for finger dexterity. Am J Occup Ther. (2003) 57:570–3. 10.5014/ajot.57.5.570 14527120

[33] Sheeran P Maki A Montanaro E Avishai-Yitshak A Bryan A Klein WMP. The impact of changing attitudes, norms, and self-efficacy on health-related intentions and behavior: A meta-analysis. Heal Psychol. (2016) 35:1178–88. 10.1037/hea0000387 27280365

[34] Hagger MS Luszczynska A. Implementation Intention and Action Planning Interventions in Health Contexts: State of the Research and Proposals for the Way Forward. Appl Psychol Heal Well-Being. (2014) 6:1–47. 10.1111/aphw.12017 24591064

[35] Quattrocchi G Greenwood R Rothwell JC Galea JM Bestmann S. Reward and punishment enhance motor adaptation in stroke. J Neurol Neurosurg Psychiatry. (2017) 88:730–6. 10.1136/jnnp-2016-314728 28377451

[36] Gardner B A. review and analysis of the use of ‘habit' in understanding, predicting and influencing health-related behaviour. Health Psychol Rev. (2015) 9:277–95. 10.1080/17437199.2013.876238 25207647 PMC4566897

[37] Swanson LR Whittinghill DM. Intrinsic or extrinsic? Using videogames to motivate stroke survivors: a systematic review. Games Health J. (2015) 4:253–8. 10.1089/g4h.2014.0074 26182071

[38] Bovend'Eerdt TJ Botell RE Wade DT. Writing SMART rehabilitation goals and achieving goal attainment scaling: a practical guide. Clin Rehabil. (2009) 23:352–61. 10.1177/0269215508101741 19237435

[39] Robinson-Smith G Harmer C Sheeran R Bellino Vallo E. Couples' coping after stroke-a pilot intervention study. Rehabil Nurs. (2016) 41:218–29. 10.1002/rnj.213 25865578

[40] Green T Haley E Eliasziw M Hoyte K. Education in stroke prevention: efficacy of an educational counselling intervention to increase knowledge in stroke survivors. Can J Neurosci Nurs. (2007) 29:13–20. 18240627

[41] Kontou E Kettlewell J Condon L Thomas S Lee AR Sprigg N. A scoping review of psychoeducational interventions for people after transient ischemic attack and minor stroke. Top Stroke Rehabil. (2021) 28:390–400. 10.1080/10749357.2020.1818473 32996432

[42] Wolf TJ Baum CM Lee D Hammel J. The development of the improving participation after stroke self-management program (IPASS): An exploratory randomized clinical study. Top Stroke Rehabil. (2016) 23:284–92. 10.1080/10749357.2016.1155278 27077987 PMC4929017

[43] Abbate M Gallardo-Alfaro L Bibiloni MDM Tur JA. Efficacy of dietary intervention or in combination with exercise on primary prevention of cardiovascular disease: A systematic review. Nutr Metab Cardiovasc Dis. (2020) 30:1080–93. 10.1016/j.numecd.2020.02.020 32448717

[44] Booth JW Neill JT. Coping strategies and the development of psychological resilience. J Outdoor Environ Educ. (2017) 20:47–54. 10.1007/BF03401002

[45] Anderson V Darling S Mackay M Monagle P Greenham M Cooper A. Cognitive resilience following paediatric stroke: Biological and environmental predictors. Eur J Paediatr Neurol. (2020) 25:52–8. 10.1016/j.ejpn.2019.11.011 31866101

[46] Gyawali P Chow WZ Hinwood M Kluge M English C Ong LK. Opposing associations of stress and resilience with functional outcomes in stroke survivors in the chronic phase of stroke: a cross-sectional study. Front Neurol. (2020) 11:230. 10.3389/fneur.2020.00230 32390923 PMC7188983

[47] Aarnes R Stubberud J Lerdal A A. literature review of factors associated with fatigue after stroke and a proposal for a framework for clinical utility. Neuropsychol Rehabil. (2020) 30:1449–76. 10.1080/09602011.2019.1589530 30905262

[48] Cao M Ferrari M Patella R Marra C Rasura M. Neuropsychological findings in young-adult stroke patients. Arch Clin Neuropsychol. (2007) 22:133–42. 10.1016/j.acn.2006.09.005 17169527

[49] Celis-Morales CA Perez-Bravo F Ibañez L Salas C Bailey MES Gill JMR. Objective vs. self-reported physical activity and sedentary time: effects of measurement method on relationships with risk biomarkers Dasgupta K, editor. PLoS ONE. (2012) 7:e36345. 10.1371/journal.pone.0036345 22590532 PMC3348936

[50] Chiu Y-L Huang S-J Lai C-H Huang C-C Jiang S-H Li S-R. Validation of self-reported smoking with urinary cotinine levels and influence of second-hand smoke among conscripts. Sci Rep. (2017) 7:15462. 10.1038/s41598-017-15526-y 29133917 PMC5684204

